# Computerized adaptive testing to screen children for emotional and behavioral problems by preventive child healthcare

**DOI:** 10.1186/s12887-020-2018-1

**Published:** 2020-03-12

**Authors:** Meinou H. C. Theunissen, Marianne S. de Wolff, Jacqueline A. Deurloo, Anton G. C. Vogels, Sijmen A. Reijneveld

**Affiliations:** 1 TNO Child Health, P. O. Box 3005, 2301 DA Leiden, the Netherlands; 2 GGD Hollands Noorden, Alkmaar, the Netherlands; 3 Department of Health Sciences, University Medical Center Groningen, University of Groningen, Groningen, the Netherlands

**Keywords:** Child mental health, Public health, Screening, Computerized adaptive testing, Preventive child healthcare

## Abstract

**Background:**

Questionnaires to detect emotional and behavioral problems (EBP) in Preventive Child Healthcare (PCH) should be short which potentially affects validity and reliability. Simulation studies have shown that Computerized Adaptive Testing (CAT) could overcome these weaknesses. We studied the applicability (using the measures participation rate, satisfaction, and efficiency) and the validity of CAT in routine PCH practice.

**Methods:**

We analyzed data on 461 children aged 10–11 years (response 41%), who were assessed during routine well-child examinations by PCH professionals. Before the visit, parents completed the CAT and the Child Behavior Checklist (CBCL). Satisfaction was measured by parent- and PCH professional-report. Efficiency of the CAT procedure was measured as number of items needed to assess whether a child has serious problems or not. Its validity was assessed using the CBCL as the criterion.

**Results:**

Parents and PCH professionals rated the CAT on average as good. The procedure required at average 16 items to assess whether a child has serious problems or not. Agreement of scores on the CAT scales with corresponding CBCL scales was high (range of Spearman correlations 0.59–0.72). Area Under Curves (AUC) were high (range: 0.95–0.97) for the Psycat total, externalizing, and hyperactivity scales using corresponding CBCL scale scores as criterion. For the Psycat internalizing scale the AUC was somewhat lower but still high (0.86).

**Conclusions:**

CAT is a valid procedure for the identification of emotional and behavioral problems in children aged 10–11 years. It may support the efficient and accurate identification of children with overall, and potentially also specific, emotional and behavioral problems in routine PCH.

## What’s new

Computerized adaptive testing adequately supports the identification of emotional and behavioral problems in children aged 10–11 years: it yields an efficient and accurate identification of emotional and behavioral problems in routine preventive child healthcare.

## Background

About 10–20% of children and adolescents have symptoms of emotional or behavioral Problems (EBP) [1]. Prevalence rates vary between the sexes depending on the type of problem [2–4]. Girls develop relatively more emotional problems during adolescence [5, 6]; boys have more behavioral problems [7]. EBP can negatively impact a child’s development and evolve into serious mental health problems in adulthood [8–10]. Early detection and treatment may considerably improve prognosis [6, 11, 12].

Many countries have preventive child healthcare (PCH) services for the early detection of health problems and EBP in children. In the United States, most children visit a pediatrician who provides pediatric primary care. In the Netherlands, PCH services provide health and developmental monitoring for all children from birth until 18 years of age. Short questionnaires are frequently used to support emotional and behavioral monitoring, e.g. the Strengths and Difficulties Questionnaire (SDQ) completed by either parents or older children [13]. PCH uses short questionnaire to limit the size of the effort requested from parents. The use of a short questionnaire by PCH may increase the number of parents that actually complete the questionnaire. However, the identification of EBP in children has proved less than satisfactory: many early EBP remain undetected [8, 14]. This problem is probably exacerbated by the limited psychometric properties of these questionnaires due to their inevitable brevity.

Computerized Adaptive Testing (CAT) is a promising alternative for the identification of EBP in community settings as it can improve the quality of early detection while remaining brief [15–19]. In a CAT, a computer selects the items to be asked based on the parent’s or child’s previous responses. A CAT procedure requires fewer items to arrive at accurate scores, and can provide the results immediately after testing. A few studies have been published on the application of Item Response Theory (IRT) models in the field of pediatric mental health care [15, 17, 18]. Devine et al. [16], Barthel et al. [15] and Hill et al. [18] showed with a (simulation) study that IRT models can be used to measure quality of life in children and adolescents. Gardner et al. (2002) [17] showed that a CAT based on the Pediatric Symptom Checklist (PSC) for parents in a simulation study can be used to measure mental health in children.

Vogels et al. (2011) [19] developed an Internet application – *Psycat* – that allows parents to use the CAT procedure to support identification of EBP in their 10–11-year-olds. In a simulation study, this Psycat was a highly sensitive and specific procedure for the identification of children with a clinical total problem score on the Child Behavior Checklist (CBCL), with an average 12 items needed to identify children with EBP [19].

Evidence is limited on whether the good validity indices of CATs in the field of pediatric mental health as assessed in simulation studies can be generalized to its use in actual practice. More specific, evidence is lacking whether the efficiency and good psychometric properties of the Psycat also regard its use in routine PCH practice. The first aim of this study was therefore to evaluate the applicability of the Psycat in routine PCH practice. This was measured as participation rate, satisfaction of participants (parents and professionals), and efficiency of the Psycat. The second aim of this study was to evaluate the validity of the Psycat in routine PCH practice, using the CBCL as criterion.

## Methods

### Sample

We obtained data on 461 children aged 10–11 years (response 41%), who were assessed during routine well-child examinations by PCH that are provided regularly for all Dutch children. We focused on the PCH examinations of children aged 10–11 years because the Psycat was developed to support identification of EBP during specifically this examination. These regarded two sub-samples:

1. *Sub-sample 1 - Application:* We obtained data about the applicability of the Psycat in a cross-sectional pilot sample in routine PCH practice. These data were collected by one public PCH organization in three towns in the south of the Netherlands, between December 2012 and March 2013. A total of 355 parents of children aged 10–11 years were invited to participate.
2. *Sub-sample 2 - Application and validation*: We obtained data from a cross-sectional sample about the applicability and validity of the Psycat in routine PCH practice. These data were collected by two public PCH organizations located in the south and west of the Netherlands, in the year 2010. A total of 762 parents of children aged 10–11 years were invited to participate.

We used both sub-samples to assess the applicability of the Psycat for parents and professionals, and only the second one to assess the validity of the Psycat. Both procedures were approved by the local institutional review board.

### Procedure and measures

The data were collected during routine well-child examinations provided to all children at ages 10–11 years. The participating PCH professionals were trained in the use of the Psycat and received a user manual. The training comprised half a day and consisted of general information about the detection of EBP in children and background information about the Psycat. Next, participants received a demo of the application and an instruction on how to interpret the results of the Psycat. A standard invitation for the PCH visit was sent to the parents, including a request for parents to complete the web-based Psycat, and additional questions. They were explicitly told that they were free to participate or not. Account details for the Psycat (username and password) were provided in the invitation for the parents. The CBCL was added to the invitation for sub-sample 2 only, and could be returned at the visit in a sealed envelope.

Parents filled out the Psycat, followed by questions on satisfaction with the Psycat. Parents also filled out the CBCL measuring EBP of their child by paper. The *Psycat*, consisted of successively offered questions on EBP out of a pool of 190 items. These questions were selected from the pool using an iterative algorithm to maximize the precision of the test, based on parent responses from the preceding questions. The item pool of the PSYCAT was based on the items from four questionnaires on EBP, i.e. the Pediatric Symptom Checklist (PSC), the SDQ, the PSYBOBA, and the CBCL [19, 20]. We separately determined Psycat outcomes for overall EBP, and for internalizing, externalizing, and hyperactivity problems.

Parent-reported *satisfaction* was measured with five questions on the use of the Psycat; responses were dichotomized, see Table 2 for response categories. *EBP* in the preceding 6 months were measured with the Dutch version of the CBCL in sub-sample 2 only. This CBCL has been shown to be highly reliable and valid [21, 22]. It comprises 120 problem items that are used to compute scores on Total problems, the broadbands Internalizing and Externalizing problems, and the CBCL DSM-derived score Hyperactivity problems. Children were allocated to a normal or clinical range according the cut-off for the 90^the^ percentile of the CBCL scores in the Dutch normative sample [21].

Professionals reviewed the Psycat results during the well-child assessments and recorded the following *background characteristics*: child gender and ethnicity. The professionals discussed the outcome of the Psycat results with the parents and the child and determined whether further actions such as more diagnostic research or a referral to (specialised) psychosocial care was needed. Furthermore, the PCH professionals reported on their *satisfaction* with the Psycat across all children that they assessed. This regarded 11 questions on a four-point scale (1 = “totally disagree”, 2 = “disagree”, 3 = “agree”, 4 = “totally agree”), which were next dichotomized (see Table 3), and an overall mark (between 1, very poor, and 10, excellent) to the Psycat.

### Analyses

We first assessed background characteristics of the sample. Next, we assessed the applicability of the Psycat by computing the participation rate, parental and professional satisfaction, and efficiency, using descriptive statistics and Cohen’s effect size index *w*. Efficiency was assessed as the mean number of items needed to identify a child with EBP. We repeated the analyses separately in each of the two subsamples to determine whether findings differed per study setting.

Third, we assessed the validity of the Psycat, using the CBCL as criterion. We calculated the percentage of children with elevated Psycat total or subscale scores. Spearman correlation coefficients were calculated to assess the overall match between Psycat subscales and the CBCL criteria in the validation dataset. The validity indices (sensitivity, specificity and Area Under Curve, AUC) of the Psycat total, externalizing, internalizing and hyperactivity problems were assessed using the corresponding CBCL scales as criteria.

## Results

### Background characteristics

A total of 1117 parents of children aged 10–11 years were invited to participate and 461 parents completed the Psycat, a participation rate of 41.3%. Differences in response by gender, age, and ethnicity were small. Participation in sub-sample 1 (only application) was much higher (68.5%) than in sub-sample 2 (28.6%) (application and validation) (*p* < 0.001, Cohen’s effect size = 0.37). Of the 461 participating parents, 450 provided complete data on the Psycat, and were included in the further analyses; of these, 181 also complete the CBCL. Sixteen PCH professionals participated and answered questions about satisfaction with the Psycat.

### Applicability: participation, satisfaction, and efficiency

As shown in Table 1, participation varied relatively little by background characteristics, being somewhat lower for non-Dutch born respondents, however. Parent satisfaction was high (Table 2). Most of the parents reported the length of the Psycat to be appropriate, and the questions to be clearly formulated. Furthermore parents reported that the questions were applicable to parental concerns and child problems. This means that if parents had, for instance concerns, the Psycat did indeed asked questions about these specific concerns. They were also positive about using a computer to answer questions about their child. Differences between the two sub-samples were very small.

**Table 1:** Applicability: Participation rate for the Psycat by background characteristics (*n* = 1117)

	Participants n (%)	Non-participants n (%)	Cohen effect size W
Child gender			0.01
Boy	229 (50.3)	309 (49.6)	
Girl	226 (49.7)	314 (50.4)	
Ethnicity			0.12
Dutch	406 (89.8)	494 (81.1)	
Migrant	46 (10.2)	115 (18.9)	
Child age			
10 years	234 (52.1)	NA^a^	
11 years	215 (47.9)		
Total	461 (41.3)	656 (58.7)	

^a^*NA* Not available

Number of missings: gender (*n* = 39), ethnicity (*n* = 56), child age (*n* = 12)

**Table 2:** Applicability: Satisfaction of parent with the Psycat (*n* = 361)

	n (%)
1. Time to complete Psycat	
(Too) long	9 (2.5)
Good	352 (97.5)
2. Clarity of the Psycat questions	
(Completely) clear	354 (98.1)
(Completely) unclear	7 (1.9)
3. Pleasant to answer questions by computer	
(Totally) positive	319 (88.4)
(Totally) negative	42 (11.6)
4. Applicability of Psycat questions about parental concerns	
No concerns	259 (71.7)
Concerns, applicable	72 (19.9)
Concerns, not applicable	30 (8.3)
5. Applicability of Psycat questions about child EBP	
No child EBP	224 (62.0)
EBP, applicable	98 (27.1)
EBP, not applicable	39 (10.8)

Due to rounding off, not all column percentages add up to 100%

Answer options: question 1 “Too long”, “long”, “good”; question 2 “Completely clear”, “clear”, “unclear”, “completely unclear”; question 3 “Totally positive”, “positive”, “negative”, “totally negative”; questions 4 and 5 “totally applicable”, “applicable”, “not applicable”, “totally not applicable”

Professionals were satisfied with the Psycat too (Table 3). They mostly reported the questionnaire to be easy, pleasant and meaningful to use, and leading to saving time because the scoring was done automatically. They considered the Psycat to be of use as a guideline to talk to parents about their children’s EBP and establish a good impression of children’s emotional and behavioral health. However, 50% of the professionals thought that the Psycat was not sufficiently clear for parents. The mean rating of the professionals for the Psycat was 6.4 (s.d. 1.3), i.e. low to moderately positive. Sub-samples were too small for separate analyses.

**Table 3:** Applicability: satisfaction of PCH professionals’ with the Psycat (*n* = 16)

	(totally) agree/total n	(%)
**Psycat…**^a^		
saves time, because scoring is done automatically	13/15	86.7
identifies ‘real’ EBP in children	12/15	80.0
fails to identify some children with EBP	2/12	16.7
classifies some children as problematic who do not actually have EBP	5/13	38.5
can be used as a conversation guide to talk with parents about children’s EBP	15/15	100
helps to establish a good picture of a child’s emotional and behavioral health	15/16	93.8
is easy to use	13/14	92.9
is pleasant to use	12/13	92.3
is meaningful to use	14/14	100
is pleasant to complete for parents	6/7	85.7
is clear for parents	4/8	50.0
How do you rate the Psycat on a scale from 1 (very poor) to 10 (very good)? (mean, sd)	6.4 (1.3)	

^a^Answer options: “totally disagree”, “disagree”, “agree” and “totally agree”

Regarding *efficiency,* the mean number of items needed in the Psycat to correctly identify a child was 15.8 (sd = 5.6) in the application-validation sample.

### Validity

Psycat total and subscale scores correlated significantly with the CBCL scores in the expected directions. The highest correlation coefficient was found between the Psycat total score and the CBCL total problems score (Spearman rho = 0.72) and the lowest one between the Psycat hyperactivity score and the CBCL internalizing score (Spearman rho = 0.26) (Table 4). About 16.3% of all children had an elevated Psycat total score, slightly higher in sub-sample 1 (20.7) than in sub-sample 2 (12.4).

**Table 4:** Validity: numbers and percentages of children with elevated Psycat scores and Spearman correlations for Psycat scores and CBCL scores (*n* = 181)

Psycat scales	Elevated Psycat scores	CBCL			
	N (%)	Total	Externalizing	Internalizing	Hyperactivity
Total	75 (16.3)	0.72*	0.59*	0.55*	0.66*
Externalizing	81 (17.6)	0.54*	0.63*	0.34*	0.43*
Internalizing	66 (14.3)	0.50*	0.30*	0.59*	0.31*
Hyperactivity	90 (19.5)	0.39*	0.32*	0.16*	0.65*

*P < 0.01

AUCs were generally high (range: 0.95–0.97) for the Psycat total, externalizing, and hyperactivity scales using corresponding CBCL scale scores as criterion (Table 5). Sensitivity varied from 0.86 to 1.00 and specificity from 0.89 to 0.93. For the Psycat internalizing scale the AUC was somewhat lower but still high (0.86). However, sensitivity was low (0.46), at rather high specificity (0.93). These validity indices (AUC, sensitivity, specificity) need to be interpreted with caution because the small number of participants who met the criterion (CBCL) leads to a low statistical power.

**Table 5:** Validity: test characteristics of the Psycat scores using elevated CBCL scores as criteria (*n* = 181)

Psycat scales	CBCL			
	Total	Externalizing	Internalizing	Hyperactivity
Sensitivity	0.86	1.00	0.46	1.00
Specificity	0.93	0.89	0.93	0.89
AUC	0.97 (0.92–1.00)	0.95 (0.91–0.996)	0.86 (0.74–0.97)	0.97 (0.94–0.995)

## Discussion

This study evaluated the applicability of the Psycat and its validity for identifying EBP in routine PCH services. Regarding applicability, participation rates were low to moderate, and most participating parents and PCH professionals were satisfied with the Psycat. Furthermore, the Psycat proved to be efficient, requiring a mean number of 16 items to correctly identify a child with EBP. Regarding validity, the Psycat was found to be valid to identify children with EBP in routine PCH care.

### Applicability: participation rate

The mean participation rate of parents was 41%, but we found differences between the two samples. The participation rate was low (29%) in the application-validation sample and satisfactory (69%) in the application sample, although both samples comprised data that was collected in a routine PCH setting. A likely explanation is that the method of data collection caused the low participation rate in the application-validation sample (29%). That method gave a large burden on parents: they had to complete the Psycat and an additional CBCL questionnaire consisting of 120 items serving as criterion, via different media (paper and computers). The much higher participation rate in the application sample suggest the Psycat as the only instrument offered to be much more acceptable. These findings suggest that the Psycat is applicable.

### Applicability: satisfaction

Most participating parents and PCH professionals were satisfied with Psycat, which aligns with other studies which also found adaptive tests to be well accepted by respondents [23–25].

Our findings indicate that professionals may underestimate the satisfaction of parents with Psycat, as they expected many parents to judge the Psycat as unclear (Table 3), while parents themselves actually reported the Psycat to be clear (Table 2). In general, these findings suggest that the Psycat is ready for further trials in routine use in PCH practice.

### Applicability: efficiency

The Psycat was an efficient procedure, requiring a mean number of 16 items to correctly identify a child, which is however four more items than in the simulation study [19]. This discrepancy can be explained in two ways. First, a simulation study may result in overfitting of the mean number of items, however this explanation is rather unlikely because separate samples were used to determine the cut-off point and to assess the validity and efficiency (mean number of items) in the simulation study. Second, this discrepancy may be due to small differences in design. Additional research is necessary to investigate this more in depth.

### Validity

We found mostly good validity indices for the Psycat in routine PCH use. However, sensitivity of the Psycat internalizing scale was low (0.46), at rather high specificity (0.93). An explanation might be that internalizing behavior are more difficult to observe for parents [26, 27]. This explanation is rather unlikely however, because the literature shows that the criterion (CBCL) for internalizing behaviors is not affected by this measurement bias [22]. A more likely explanation is the small number of participants who met the criterion (CBCL), leading to a low statistical power of our validation study in this respect. As a consequence, the validity indices from this study on emotional problems need to be interpreted with caution.

The mostly good validity indices for the Psycat in routine PCH confirm the findings of the previous simulation study regarding the Psycat of Vogels et al. [19] and other (simulation) studies regarding CATs aimed to measure quality of life [16, 18] or mental health [17] in the field of pediatric mental health care. Gardner’s CAT assessed the same construct (mental health in children) as the Psycat. Gardner et al. [17] showed that CAT based on the Pediatric Symptom Checklist (PSC) for parents in a simulation study reproduced the results of the full scale PSC with greater efficiency. There are however some differences with respect to the Psycat. Gardner used the full PSC as criterion for EBP, while the Psycat used the widely validated CBCL. Furthermore, Gardner used only PSC items to develop the CAT, we use items from four questionnaires. Lastly, the Psycat was studied in routine PCH, whereas Gardner study was limited to a simulation study. Our study therefore provides a stronger argument for the validity and feasibility of CAT-based procedures in the field of mental health of children.

### Strengths and limitations

Our study has a number of strengths, such as its community-based nature and embedding in routine PCH practice. Moreover, we were able to assess various aspects of applicability, including the parent- and the professional perspective.

However, some limitations should also be considered. First, we used the very well-validated CBCL questionnaire as the validation criterion, and not clinical assessments such as psychiatric interviews. Clinical assessments may provide additional information, but could not be used in this study because of their complexity and high costs. Second, the participation rate regarding the validation was low, which may have led to selective non-response among parents of children with EBP. This may affect prevalences and perhaps also validity indices. Finally, some of the items predicting the criterion were part of the criterion itself, i.e. the CBCL items. However, this regards relatively few of the items included in the CAT and it has a small effect on conclusions regarding validity.

### Implications

Our findings indicate that the Psycat is an efficient instrument that could be validly used to help parents and professionals identify EBP in routine PCH settings. These promising findings suggest that the Psycat is suitable for further trials in routine use in PCH services. It provides an efficient and valid procedure for the assessment of EBP, overall and more specific, for routine use in PCH.

Our study also showed that parents in a real-life setting needed more items to identify a child with EBP than they needed in a simulation setting. This implies that simulation studies of adaptive tests may provide an over-optimistic picture of its performance in real-life healthcare settings. This latter aspect and the low to moderate response rates definitely require additional research on the performance of CAT in both PCH services and other healthcare settings.

## Conclusions

Our findings indicate that the Psycat is a feasible, efficient and valid (accurate) procedure to identify EBP in 10–11 years old children. The Psycat may improve the identification of EBP in PCH practice. Further research on the validity and implementation of CAT in routine PCH may largely add to efficient and high-quality preventive care.

## Data Availability

The datasets used and/or analyzed during the current study are available from the corresponding author on reasonable request.

## Abbreviations

AUC: Area Under Curves
CAT: Computerized Adaptive Testing
CBCL: Child Behavior Checklist
EBP: Emotional and Behavioral Checklist
IRT: Item Response Theory
PCH: Preventive Child Healthcare
PSC: Pediatric Symptom Checklist
SDQ: Strengths and Difficulties Questionnaire
